# Neurodevelopmental disorder with microcephaly, hypotonia, and variable brain anomalies in a consanguineous Iranian family is associated with a homozygous start loss variant in the PRUNE1 gene

**DOI:** 10.1186/s12920-022-01228-6

**Published:** 2022-04-04

**Authors:** Mehdi Agha Gholizadeh, Mina Mohammadi-Sarband, Fatemeh Fardanesh, Masoud Garshasbi

**Affiliations:** 1 Department of Medical Genetics, Faculty of Medical Sciences, Tarbiat Modares University, Tehran, Iran; 2 Department of Medical Genetics, DeNA Laboratory, Tehran, Iran; 3 Shemiranat Genetic Counselling Center, State Welfare Organization, Tehran, Iran; 4 PardisGene Company, Tehran, Iran

**Keywords:** *PRUNE1*, Neurodevelopmental disorder, NMIHBA, Whole-exome sequencing

## Abstract

**Background:**

Homozygous or compound heterozygous *PRUNE1* mutations cause a neurodevelopmental disorder with microcephaly, hypotonia, and variable brain malformations (NMIHBA) (OMIM #617481). The *PRUNE1* gene encodes a member of the phosphoesterase (DHH) protein superfamily that is involved in the regulation of cell migration. To date, most of the described mutations in the *PRUNE1* gene are clustered in DHH domain.

**Methods:**

We subjected 4 members (two affected and two healthy) of a consanguineous Iranian family in the study. The proband underwent whole-exome sequencing and a start loss identified variant was confirmed by Sanger sequencing. Co-segregation of the detected variant with the disease in family was confirmed.

**Results:**

By whole-exome sequencing, we identified the a start loss variant, NM_021222.3:c.3G>A; p.(Met1?), in the *PRUNE1* in two patients of a consanguineous Iranian family with spastic quadriplegic cerebral palsy (CP), hypotonia, developmental regression, and cerebellar atrophy. Sanger sequencing confirmed the segregation of the variant with the disease in the family. Protein structure analysis also revealed that the variant probably leads to the deletion of DHH (Asp-His-His) domain, the active site of the protein, and loss of *PRUNE1* function.

**Conclusion:**

We identified a start loss variant, NM_021222.3:c.3G>A; p.(Met1?) in the *PRUNE1* gene in two affected members as a possible cause of NMIHBA in an Iranian family. We believe that the study adds a new pathogenic variant in spectrum of mutations in the *PRUNE1* gene as a cause of *PRUNE1*-related syndrome.

## Background

Neurodevelopmental disorder with microcephaly, hypotonia, and variable brain anomalies (NMIHBA) (OMIM#617481) is a rare autosomal recessive disorder characterized by hypotonia, progressive microcephaly, plagiocephaly, spastic quadriparesis, global developmental delay, intellectual disability, and optic atrophy. Brain imaging in these patients shows cortical atrophy, thin corpus callosum, cerebellar hypoplasia, and delayed myelination [1]. NMIHBA is caused by homozygous or compound heterozygous mutations in the *PRUNE1* gene at 1q21.3. This gene encodes an exopolyphosphatase member of the DHH (Asp-His-His) protein, which are the enzymes that break phosphoester bonds in substrates [2]. Although *PRUNE1* is expressed in human adult tissues, it is highly expressed in early embryonic and fetal stages and is indicated to have an important role in the developing human brain [3]. Further, it has a crucial role in microtubule polymerization, cell migration, cell differentiation, and proliferation [1, 4]. To date, several mutations have been reported in *PRUNE1*, most of which are clustered in DHH domain. The DHH domain contains highly-conserved charged residues required for enzymatic function and substitutions in any of its three amino acids are predicted to result in a loss of function [5].

Herein, by performing whole-exome sequencing, we identified a start loss variant, NM_021222.3:c.3G>A; p.(Met1?); in the *PRUNE1* gene. The variant was confirmed by Sanger sequencing in the patient and both parents. Furthermore, Sanger Sequencing results demonstrated co-segregation of the c.3G>A variant with the disease in this family. This report provides a pathogenic variant in the *PRUNE1* gene as a cause for the *PRUNE1*-related syndrome by affecting the Methionine start codon.

## Methods

### Ethical consideration

The informed consent was taken from all subjects. All clinical information and medical histories were collected at the Genetic Counselling Center of State Welfare Organization and the Department of Medical Genetics, DeNA laboratory, Tehran, Iran. Ethics Committee of Children's Medical Center Hospital, Tehran, Iran approved the study. The family also provided written informed consent for publication of their pertinent data included in this paper.

### Subjects

**Fig. 1:**
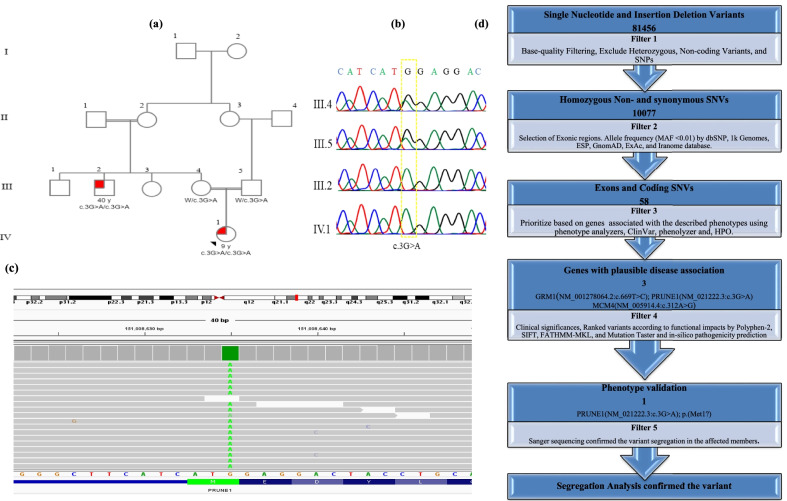
Pedigree and chromatograms in the family. **a** The pedigree is comprised of four generations. The black arrow shows the proband of the family. The variant, c.3G>A in *PRUNE1*, has been demonstrated that segregated in this family. **b** Sequence chromatogram showing a heterozygous and homozygous state of c.3G>A variant in PRUNE1 in the proband's parents (III:4; III:5) and the patients (III:2; IV:1), respectively. **c** Integrative Genomics Viewer of the genome sequencing revealed a homozygous state of c.3G>A variant in the patient (IV:1). **d** Schematic representation of filtering strategies exploited in this study

We enrolled 4 members of the family in our study (two affected and two healthy) (Fig. 1a). The consanguineous family, originating from Tehran province of Iran, was suspected of Hereditary Spastic Paraplegia (HSP). The proband underwent whole-exome sequencing and the affected subject (III:2 and IV:1) were adjusted by medical records including a general physical examination, neuroimaging, karyotyping, and other conventional biochemical tests.

### Sample collection and DNA extraction

DNA was extracted from the peripheral blood of all family members using the High Pure PCR template preparation kit (Roche: Product No. 11814770001). Pedigree is shown in Fig. 1a. Thence, the quality and quantity of the extracted DNAs were measured by Thermo Scientific™ Nanodrop 2000 (Thermo Fisher Scientific).

### Whole exome sequencing

Whole Exome Sequencing (WES) analysis was performed for the proband (IV:1). The Twist Human Core Exome Plus kit was used to enrich approximately 36.5 Mb of the Human Exome from fragmented genomic DNA. The generated library was sequenced on an Illumina Hiseq 4000 platform to obtain an average coverage depth of 100X. All in all, 98% of the targeted bases were covered with at least 20X.

### In-silico bioinformatics analysis

**Fig. 2:**
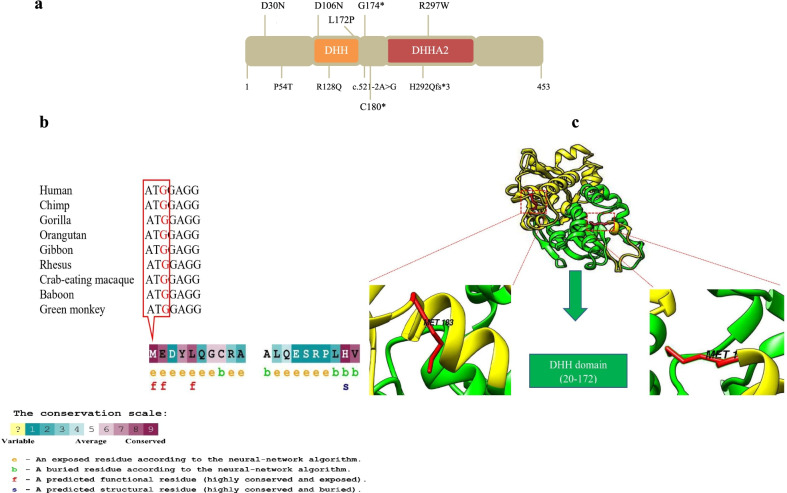
**a** Pathogenic variants in PRUNE1 identified in NMIHBA patients reported to date. The majority of pathogenic variants cluster in the DHH domain. **b** The amino acid residues of *PRUNE1* are colored based on conservation scores by the ConSurf database. UCSC and Consurf database demonstrate evolutionary conservation in nucleotide and protein levels of the variant site, c.3G>A; p.(Met1?), respectively. **c** The three-dimensional structure of *PRUNE1* is shown. The picture was delineated by using UCSF Chimera (v:1.15). Protein structure reveals the translation can be initiate at a downstream site at codon 183 would cause the deletion of the DHH domain

An in-house bioinformatics pipeline, including base calling, adapters trimming, FASTQ file quality controls, alignment of reads to GRCh37/hg19 genome assembly, primary filtering out of low-quality reads and probable artifacts, and subsequent variant calling and annotation were applied. All disease-causing variants reported in HGMD® [6], ClinVar [7] as well as all variants with minor allele frequency (MAF) below 1% in gnomAD (https://gnomad.broadinstitute.org), ExAC (Exome Aggregation Consortium) (http://exac.broadinstitute.org/) database, and 1000 genome project (http://www.1000genomes.org/), were considered. The relevant variants in coding exons and flanking ± 20 intronic bases were considered. The probable effects of pathogenicity/prioritization of candidate variants were predicted using SIFT [8], PROVEAN [9], PolyPhen-2 [10], and MutationTaster [11] (Table 2). All probable pathogenic variants were double-checked in HGMD® [6] and ClinVar [7]. Finally, the frequency of the variants was checked in the Iranome as a local database (http://www.iranome.ir). ConSurf server (http://consurf.tau.ac.il/2016/) [12] and UCSC database [13] were applied to provide an evolutionary conservation profile for PRUNE1 protein and DNA sequence, respectively (Fig. 2b). Furthermore, the protein structure and possible effects of the start loss variant on *PRUNE1* were analyzed by UCSF Chimera software after building the PDB structure file based on the SWISS-PROT [14] and Phyre2 [15] (Fig. 2c).

### Variant validation and co-segregation analysis

Sanger sequencing was employed to validate the detected variant in the proband and her parents, and then co-segregation analyses were performed to discern whether the causative homozygous variant in *PRUNE1* segregates with the disease phenotype or not. The primers were designed by Primer3.0 (http:// bioinfo.ut.ee/primer3-0.4.0) web-based server (Table 1). We check out the 3′ ends of primers for the lack of SNPs using the dbSNP database. The primers specificity was checked by the in-silico-PCR tool in UCSC genome browser (https://genome.ucsc.edu/cgi-bin/hgPcr) and Primer blast of NCBI genome browser (https://www.ncbi.nlm.nih.gov/tools/ primer-blast/). Consequently, polymerase chain reaction (PCR) was utilized in standard conditions [16] and the PCR products were sequenced on an ABI 3130 with the ABI PRISM BigDye Terminator v. 3.1 sequencing kit (Applied Biosystems, USA). The Sequences were analyzed using Genome Compiler online tool (http://www.genomecompiler.com/) and Mutation Surveyor v.3.24 (SoftGenetics) to identify the alternations (Fig. 1b). Variants were annotated based on the standards of the Human Genome Variation Society (HGVS) nomenclature [17].

**Table 1:** Sequences of the primers used to validate the variant by Sanger sequencing

Gene	Variant	Primers
*PRUNE1*	NM_021222.3:c.3G>A p.(Met1?)	Forward 5'ATTCGTCGGGGAAACCTCT 3' Reverse 5'CTAAACTGGCTTCTCGCTCCT 3'

## Results

### Clinical findings

The proband (IV:1) was a 9-year-old girl referred to Genetic Counselling Center of State Welfare Organization, Tehran, Iran due to hypotonia. She was the first child born to a consanguineous marriage. She was born via Cesarean delivery (C-section) due to breech presentation unexpectedly. Her weight was 3.750 kg at birth (~ 75% percentile). At the age of 9 years, her head circumference was 51 cm (~ 25% percentile), which indicates that she does not have microcephaly. According to her parents, following her sixth month of vaccination, rashes had appeared on all of her body skin and her symptoms intensified. The first symptom was swallowing problem (dysphasia). She has been hospitalized four times for aspiration pneumonia so far. According to her mother, after visited by ophthalmologist at 6 years old, one of the eyes was diagnosed to suffer from optic nerve problem. Moreover, she showed developmental regression at that age. At the age of 8, she developed some problems such as severe spastic quadriplegic cerebral palsy (CP), central hypotonia, dysphasia, and spastic seizure. Due to severe spasticity, she had experienced twice hip dislocation then. Severe spasticity mainly affected the lower limbs more than the upper limbs. Over the following years, she had swallowing and speech difficulties but reacted to words normally. She is unable to hold her head still. Furthermore, she has shown hyperreflexia and sever scoliosis. All general tests of the patient, including Complete Blood Count and blood chemistry, were normal. Furthermore, serum ammonia, serum creatine kinase, thyroid function, blood lactate, blood ammonia, serum copper, blood and urinary amino acids, urine organic acids, urea cycle metabolism, B-oxidation of fatty acids metabolism, and very-long-chain fatty acids were all normal.

In Brain magnetic resonance imaging (MRI) findings, Ventricles were normal and no sign of hydrocephalus was noted. Basal ganglia, sellar, parasellar, and pituitary regions were normal. The gray and white matter differentiation was well maintained. There was no evidence of infarct, hemorrhage, intraparenchymal mass, or midline shift. Atrophy of the cerebellar vermis was seen at 9 months of age.

Furthermore, patient III:2 had similar symptoms to the proband such as hypotonia, intellectual disability, speech disorder, and cerebral palsy. Genetic counseling revealed a positive history of intellectual disability in 2 affected members in this family, however, no medical records were available.

### Mutation analysis

In total, 81,456 variants were found by WES in the proband after alignment and SNV calling. By excluding the variants with allele frequency greater than 1% in dbSNP, 1000 Genomes Project, Exome Sequencing Project (ESP), GnomAD, ExAc, and Iranome databases, prioritizing according to their functional impacts and choosing genes with plausible disease association using phenotype analyzers, ClinVar, phenolyzer [18], and Human phenotype ontology [19], only three variants remained. These variants were then prioritized according to their functional impacts by Polyphen-2, SIFT, FATHMM-MKL, and Mutation Taster. By considering clinical features of the patients, two of these variants (NM_001278064.2:c.669T>C; p.(Asn223Asn) in GRM1 gene; NM_005914.4:c.312A>G; p.(Arg104Arg) in MCM4 gene) excluded and consequently, based on the clinical phenotypes of patients and in-silico pathogenicity prediction, a start loss variant, NM_021222.3:c.3G>A; p.(Met1?) (ClinVar accession SCV002061319) in *PRUNE1* was identified. The details of the filtration steps are presented in Fig. 1d. Eventually, the samples from the available members of the family (III:2, III:4, and III:5) were subjected to Sanger sequencing, and co-segregation of the detected variant with the disease in family was confirmed (Fig. 1b). Integrative Genomics Viewer (IGV) screenshot showed a homozygous state of c.3G>A variant in the proband (Fig. 1c). We also classified the variant based on the American College of Medical Genetics and Genomics Association for Molecular Pathology (ACMG-AMP guidelines) (http://wintervar.wglab.org) into a likely Pathogenic Variant. Several prediction tools such as Mutation Taster, FATHMM-MKL, PolyPhen-2, and SIFT suggested that this variant is deleterious to protein function; Table 2. Genes related to the clinical phenotype of the patients in HPO terms applied in the filtration process included Seizures (HP:0001250), Spastic tetraplegia (HP:0002510), Hypotonia (HP:0001252), Cerebellar vermis atrophy (HP:0006855), Developmental regression (HP:0002376), Dysphagia (HP:0002015). Also, conservational study using UCSC and Consurf revealed that the variant is located in a highly conserved area in nucleotide and protein levels, respectively (Consurf score ~ 9.0).

**Table 2:** In silico prediction used to confirm the pathogenicity of the identified variant in *PRUNE1*

Variant	Gene/genomic Position (hg19)	Zygosity											
		Patient	Mother	Father	MutationTaster	EXACT	SIFT	PROVEAN	PolyPhen-2	Iranome	FATHMM-MKL	Varsome	ACMG Classification
c.3G>A; p.(Met1?)	*PRUNE1* (chr1-150981111-G-A)	Hom	Het	Het	Disease causing	–	Damaging	Neutral	Probably Damaging	–	Damaging	Pathogenic	Likely pathogenic

## Discussion

The *PRUNE1* gene, located on chromosome 1q21.3, encodes a 453 amino acid protein, that is, highly expressed in the human fetal brain and involved in the regulation of neuronal migration and proliferation [4]. It harbors 2 domains DHH (20–172 amino acids) and DHHA2 (215–359 amino acids). The DHH super-family can be categorized into two main groups based on their C-terminal motif which is highly conserved within each group. Members of this superfamily have four other motifs that are predicted to be responsible for binding metal cofactors and enzymatic function [20]. Most of the described mutations in the *PRUNE1* gene so far are clustered in the DHH domain. PRUNE through interaction with glycogen synthase kinase-3 (GSK-3B), a suppressor of neurite outgrowth, synapse formation, and neurogenesis might play a molecular role in brain development [21]. Furthermore, PRUNE in a complex interaction network by regulating cellular mobility and stimulating expression of genes involved in metastatic pathways, is associated with cancer metastasis [22, 23]. Homozygous or compound heterozygous mutations in the *PRUNE1* gene cause the neurodevelopmental disorder with microcephaly, hypotonia, and variable brain anomalies (NMIHBA) (OMIM#617481) that is characterized by global developmental delay, microcephaly, central hypotonia, spastic quadriplegia, and cerebral and cerebellar atrophy [1].

By WES, we identified a start loss variant, NM_021222.3:c.3G>A; p.(Met1?), in the *PRUNE1* in an Iranian female patient presenting spastic quadriplegic cerebral palsy (CP), central hypotonia, developmental regression, and seizure. Since a start codon mutation is likely to alter the translation initiation codon, the detected variant, p.Met1?, is predicted to abolish translation of the *PRUNE1* polypeptide. In this case, the translation will either initiate at a downstream site, or not at all. According to a 3D model of PRUNE, the next closest in frame ATG is at codon 183, thus even if this ATG served as an initiation codon, the start of translation at this site would cause the deletion of the DHH domain from the N-terminus of the mature *PRUNE1* and likely to lead to loss of function of PRUNE (Fig. 2c). To date, most of the pathogenic variants in NMIHBA patients are reported in DHH domain (p.Asp106Asn, p.Arg128Gln, p.Gly174* and c.521-2A>G in DHH domain and p.His292Glnfs*3 and p.Arg297Trp in DHHA2 domain) [1, 3, 24–28] (Fig. 2a). The truncating mutations result in lack of the domains and affect either or both of the catalytic DHH or DHHA2 domains and might lead to decreased PRUNE activity.

Homozygous start loss variant, NM_021222.3:c.3G > A; p.(Met1?) in *PRUNE1* gene identified in this study, probably leads to the deletion of DHH (Asp-His-His) domain, the active site of the protein, and loss of *PRUNE1* function. A change in any of these three amino acids has been indicated to greatly reduce the enzyme’s activity [5]. A homozygous variant p.Asp106Asn, which was previously reported in Turkish and Italian patients with microcephaly, spastic quadriparesis, hypotonia, cerebellar atrophy, and neurodevelopmental impairment, altered one of these three conserved amino acids DHH (Asp-His-His) domain [3]. Additionally, compound heterozygous (c.G383A; p.R128Q and c.G520T; p.G174X) variants in two affected siblings from the United States with severe developmental delay, regression, seizures, and microcephaly have been reported [3]. The main characteristics of these patients are similar to our patients’ phenotypes, but most of the reported patients had progressive microcephaly which did not present in our patient. Similar to over case, in another study, a patient with a homozygous (c.540T>A; p.Cys180*) *PRUNE1* variant has been reported who did not exhibit progressive microcephaly [29]. Furthermore, Costain et al. revealed a homozygous likely pathogenic variant in *PRUNE1* (c.521-2A>G: IVS4-2A>G; NM_021222.1), affecting the catalytic DHH domain, in a patient with global developmental delay, infantile spasms, and central hypoventilation without microcephaly [27]. Thus, it can be suggested that p.(Met1?) may lead to the lack of DHH domain and in turn reduction or absence of the protein activity. Remarkably, the start loss variant (NM_021222.3: (PRUNE1): c.3G>A (p.Met1Ile)) was submitted to the ClinVar database on Jul 15, 2021, as likely pathogenic (ClinVar accession: SCV001759980).

Additionally, we accomplished a clinical and literature review of all 41 NMIHBA patients reported to date in order to further convey the phenotypic spectrum of NMIHBA (Table 3). Cardinal features the majority of patients include developmental delay DD (87.80%), Intellectual disability (82.93%) with speech disorders (75.61%) and cerebral and cerebellar atrophy (48.78%). Hypotonia (41.46%), spastic quadriplegia (21.95%), microcephaly (60.98%), and seizures (58.54%) are features observed in a sizable number of patients. Radiological results display that most of the patients have delayed myelination, thin corpus callosum, and white matter abnormality, which is not reported in our cases. So, in view of previous reports the clinical severity of the phenotype was variable even across patients harboring the same variant allele. No relationship between presence/absence of particular clinical features with the various mutations was obviously observed. Further reporting of patients with novel mutation enables a better understanding of the phenotypic spectrum and helps in advising newly diagnosed patients.

**Table 3:** Clinical characteristics of 41 patients with *PRUNE1* mutations

*PRUNE1* mutation	Ethnicity	Age at evaluation	Sex F/M	Birth weight	OFC at birth	Speech disorder	Microcephaly	ID	Spastic quadriplegia	Neuromuscular finding	Seizures	Metabolic profile	Dysphasia	DR	DD	MRI findings	EEG findings	Study
Homozygous p.(Met1?)	Iranian	9 yo	F	3750 cm	NA	Yes	No	No	Yes	Hypotonia	Yes	Unremarkable	Yes	Yes	No	Cerebellar atrophy	NA	This study
	Iranian	40 yo	M	NA	NA	Yes	No	Yes	Yes	Hypotonia	Yes	Unremarkable	NA	Yes	No	Cerebellar atrophy	NA	
Compound heterozygous p.(Asp106Asn) and p.(Cys180*)	Japanese	12 mo	F	2802 g	31.2 cm	NA	NA	Yes	Yes	Hypotonia	No	Unremarkable	NA	NA	Yes	cerebral and cerebellar atrophy, thin corpus callosum, white matter changes, and abnormal signal intensity of the brainstem	Hypsarrhythmia	[30]
Homozygous (13 patients) p.(Asp30Asn), p.(Pro54Thr), p.(Arg297Trp), p.(Asp106Asn)	Omani, Iranian, Italian and Indian	0.3 − 21.0 yo	9 F; 4 M	NA	NA	Yes (13/13)	Yes (13/13)	Yes (13/13)	NA	NA	Yes (6/13)	NA	NA	NA	Yes (13/13)	Delayed myelination (5/13), wide spread white matter hypodensity or abnormalities (4/13), Cerebral/cerebellar atrophy (3/13), thin corpus callosum (2/13)	NA	[1]
Homozygous (5 patients) p.(Asp30Asn), p.(Asp106Asn), Compound heterozygous p.(Arg128Gln) and p.(Gly174 ∗)	Saudi; Turkish; US	1.5 − 5.5 yo	2F, 3 M	NA	NA	Yes (3/5)	Yes (5/5)	Yes (5/5)	Yes (2/5)	Hypotonia (2/5)	NA	NA	NA	NA	Yes (5/5)	Hypomyelination (2/5), Cerebral atrophy (5/5), Cerebellar atrophy (5/5), Thin corpus callosum (5/5)	NA	[3]
Homozygous (12 patients) p.(Leu172Pro), p.(Asp106Asn), g.150984457-151016662del (Ex2-8 Del)	Lebanese; Turkish; European; North African	0 m − 12 yo	10 F; 2 M	NA	− 2.96 to + 2 s.d	Yes (10/12)	Yes (2/12)	Yes (11/12)	NA	Hypotonia (5/12)	Yes (11/12)	NA	Yes (11/12)	NA	Yes (10/12)	Cerebral atrophy (7/12), Cerebellar atrophy (6/12); Hyperintense brain lesions (4/12)	Focal spasms (2/12); Slow multifocal spikes (2/12)	[31]
Homozygous p.(Asp106Asn)	Italian	9 mo	NA	NA	NA	NA	NA	NA	NA	Hypotonia	NA	mild CK increase (976 U/L, n.v. < 150)	NA	NA	NA	cortical atrophy, severe thinning of white matter, and signal changes in the periventricular white matter and pons	multifocal epileptic abnormalities	[28]
Homozygous p.(Arg128Gln)	Saudi Arabia	12 mo	F	2700 g	33.0 cm	Yes	Yes	NA	Yes	Hypotonia	No	Unremarkable	NA	NA	Yes	delayed myelination, slightly abnormal shape of the corpus callosum, and mild frontal cerebral atrophy	NA	[25]
	Saudi Arabia	12 mo	F	2000 g	33.0 cm	NA	Yes	NA	Yes	Hypotonia and appendicular spasticity with deep tendon reflexes + 3	No	NA	NA	NA	Yes	A slightly abnormally shaped corpus callosum and slightly prominent CSF spaces anteriorly with normal myelination	NA	
Homozygous p.(Cys180*)	Japanese	7 yo	F	NA	31.0 cm	NA	No	Yes	Yes	Hypotonia	Yes	NA	NA	NA	Yes	Delayed myelination; Cerebral atrophy; Cerebellar atrophy	Hypsarrhythmia	[29]
Homozygous c.521-2A>G	Canadian provinces	2 yo	M	5180 g	38.0 cm	NA	No	NA	NA	Hypotonia	Yes	NA	NA	No	Yes	cortical atrophy and a small cerebellum, thinning of the corpus callosum, and patchy T2 hyperintensity in the cerebral white matter	Hypsarrhythmia	[27]
Homozygous c.874_875insA p.(H292Qfs*3)	Turkish	3 yo	M	3750 g	36.0 cm	NA	Yes	NA	Yes	Hypotonia	Yes	NA	NA	NA	Yes	cerebral and cerebellar atrophy with delayed myelination and inferior vermis hypoplasia	NA	[26]
Compound heterozygous p.(Arg128Gln) and p.(Gly174 ∗)	European	4 yo	F	15,100 g	41.5 cm	Yes	Yes	Yes	No	Hypotonia	Yes	NA	Yes	No	Yes	Moderate/severe progressive global brain atrophy; cerebral and cerebellar atrophy	Background slowing, infrequent temporal spike waves	[32]
	European	20 mo	M	1280 g	41.0 cm	Yes	Yes	Yes	No	increased limb tone, brisk tendon reflexes	Yes	NA	Yes	No	Yes	Mildly prominent lateral ventricles and sulci, thinner splenium and corpus callosum	Modifiedhypsarrythmia, multifocalepileptiformdischarges	

ID: Intellectual disability, DD: Developmental delay; DR: Developmental regression

To conclude, by using targeted-exome sequencing, we identified a start loss variant, NM_021222.3:c.3G>A; p.(Met1?) in the *PRUNE1* gene in two affected members as a possible cause of NMIHBA in an Iranian family. The putative variant meets the criteria of being pathogenic, but we strongly recommend doing functional analysis in order to further confirm the molecular mechanism of the pathogenesis of this variant. We believe that the identification of novel variants in *PRUNE1* may also be helpful in the diagnosis of *PRUNE1*-related syndrome.

## Data Availability

The datasets generated during and/or analysed during the current study are available from the corresponding author on reasonable request. Human variant and phenotypes have been reported to ClinVar (accession number: SCV002061319) and available at https://www.ncbi.nlm.nih.gov/clinvar/submitters/508426. Whole exome sequencing data for the patient (IV:1) is available in the NCBI SRA under the accession numbers PRJNA806464 (https://www.ncbi.nlm.nih.gov/sra/?term=PRJNA806464).

## Abbreviations

NMIHBA: Neurodevelopmental disorder with microcephaly, hypotonia, and variable brain anomalies
ACMG: American College of Medical Genetics
OMIM: Online mendelian inheritance in man
WES: Whole-exome sequencing
MAF: Minor allele frequency
MRI: Brain magnetic resonance imaging
